# Profiling Autism and Attention Deficit Hyperactivity Disorder Traits in Children with SYNGAP1-Related Intellectual Disability

**DOI:** 10.1007/s10803-023-06162-9

**Published:** 2023-12-06

**Authors:** Damien Wright, Aisling Kenny, Lindsay A. M. Mizen, Andrew G. McKechanie, Andrew C. Stanfield

**Affiliations:** Patrick Wild Centre, Division of Psychiatry, Kennedy Tower, Royal Edinburgh Hospital, University of Edinburgh, EH10 5HF Edinburgh, UK

**Keywords:** SYNGAP1-related intellectual disability, Autism, ADHD, Intellectual disabilities

## Abstract

SYNGAP1-related ID is a genetic condition characterised by global developmental delay and epilepsy. Individuals with SYNGAP1-related ID also commonly show differences in attention and social communication/interaction and frequently receive additional diagnoses of Autism Spectrum Disorder (ASD) or Attention Deficit Hyperactivity Disorder (ADHD). We thus set out to quantify ASD and ADHD symptoms in children with this syndrome. To assess ASD and ADHD, parents and caregivers of a child with SYNGAP1-related ID (N = 34) or a typically developing control (N = 21) completed the Social Responsiveness Scale-2, the Social Communication Questionnaire with a subset of these also completing the Conners-3. We found that those with SYNGAP1-related ID demonstrated higher levels of autistic traits on both the SRS and SCQ than typically developing controls. On the SRS, those with SYNGAP1-related ID scored highest for restricted repetitive behaviours, and were least impaired in social awareness. On the Conners-3, those with SYNGAP1-related ID also showed a high prevalence of ADHD traits, with scores demonstrating difficulties with peer relations but relatively low occurrence of symptoms for DSM-5 conduct disorder and DSM-5 oppositional defiant disorder. Hierarchical clustering analysis highlighted distinct SYNGAP1-related ID subgroups for both ASD and ADHD traits. These findings provide further characterisation of the SYNGAP1-related ID behavioural phenotype, guiding diagnosis, assessment and potential interventions.

**Supplementary Information:**

The online version contains supplementary material available at 10.1007/s10803-023-06162-9.

Attention-Deficit/Hyperactivity Disorder (ADHD) and Autism Spectrum Disorder (ASD) are neurodevelopmental conditions that are reported to be more common in a number of genetic forms of intellectual disability (ID). One such genetic disorder, SYNGAP1-related Intellectual Disability (henceforth referred to as SYNGAP1-ID), has been reported in case series to carry high rates of diagnosis of ASD and, possibly, ADHD (Holder et al., 2019; Mignot et al., 2016). However, it is currently unclear if there is a specific profile of ASD and ADHD for children with SYNGAP1-ID, and what this may look like.

SYNGAP1-ID is estimated to account for up to 1% of ID cases, making it potentially one of the most common genetic causes of ID, alongside other syndromes such as fragile X syndrome. It is caused by a de novo loss of function mutation in the *SYNGAP1* gene, which disrupts the Synaptic GTPase-activating protein (SYNGAP). The SYNGAP protein is vital in dendritic spine maturation and synaptic plasticity. Reports have highlighted most individuals have truncating variants, however missense and microdeletions have also been described (Mignot et al., 2016; Writzl & Knegt, 2013; Zollino et al., 2011). SYNGAP1-ID is characterised by developmental delay, epilepsy, intellectual disability, sleep disturbances and behavioural issues. In particular, social impairments have been commonly identified in SYNGAP1-ID*.* ASD features have been described in those with SYNGAP1-ID, with a reported prevalence ranging from around 50 to 73% (Jimenez-Gomez et al., 2019; Mignot et al., 2016). In addition, issues with inattention have also been described in this population (Holder et al., 2019).

ASD is a heterogeneous neurodevelopmental condition that is defined in Diagnostic Statistical Manual-5 (DSM-5) and International Classification of Disease-11 (ICD-11), the two most widely used international diagnosis classifications, by significant differences in social interaction/communication and restricted/repetitive patterns of behaviour and interest. Social communication and interaction traits can include differences in social overtures, reduced sharing of interests/emotions, differences in eye contact, and a more limited range of verbal and non-verbal communication. Restricted and repetitive behaviours are often manifested in behaviours such as simple motor stereotypies, insistence on sameness, distress (which can be extreme) at small changes, and rigid thinking patterns. By definition, the onset of ASD is early in childhood, is lifelong in nature, and is estimated to occur in up to 2.3% of the general population (Hirota & King, 2023).

Although no one specific cause of ASD has been identified, genetic factors are considered to play a strong role (Abrahams & Geschwind, 2008) and the presence of ASD and broader autistic features have been described in a number of genetic syndromes including fragile X syndrome (FXS), Angelman and Cornelia de Lange syndromes (Moss et al., 2013). However, the prevalence and profile of ASD varies across genetic syndromes (Lesniak-Karpiak et al., 2003; Moss & Howlin, 2009; Richards et al., 2015; Trillingsgaard & Østergaard, 2004). The diagnosis of ASD in genetic syndromes is further complicated by ID, with the general trend being that the more profound the intellectual disability, the greater the likelihood that they will be diagnosed with ASD (Skuse, 2007). Reports have suggested that ASD occurs in up to approximately 40% of individuals with severe to profound levels of intellectual disability (La Malfa et al., 2004).

ADHD is a common condition characterised by symptoms of hyperactivity, inattention, impulsivity and impaired executive function. Based on the DSM-5 criteria, children aged up to 16 years old must display six inattention and/or six hyperactivity/impulsivity symptoms, present for at least 6 months, which are disruptive and impair functioning. The prevalence of ADHD is reported to range from 2.9% in children, whilst for adults in the general population it is around 4% (Sacco et al., 2022; Song et al., 2021). Typically, it is onsets in childhood and as such, ADHD is associated with a number of adverse outcomes including social and educational impairment during school age years (Harpin et al., 2016). Untreated, it can potentially lead to the development of further issues throughout adolescence and adulthood such as conduct and personality disorders. ADHD frequently co-occurs not only with ASD but also epilepsy and childhood mental health problems, especially anxiety.

Little is known about the presentation of ADHD in individuals with intellectual disabilities as often they are excluded from ADHD studies, even though it has long been acknowledged that those with lower IQ are more likely to present with ADHD symptoms (Simonoff et al., 2007). In those with ID, ADHD prevalence rates are suggested to be higher than those without ID, affecting as high as around 14% of individuals (Dekker & Koot, 2003). Despite this, ADHD in those with ID may actually be underdiagnosed due to diagnostic overshadowing. High rates of ADHD have been reported in genetic conditions with developmental delay including FXS, Klinefelter’s, Turner Syndrome, and Williams Syndrome (Lo-Castro et al., 2011), with the presentation of ADHD-related symptoms varying between disorders.

At present, the clinical presentation and degree of ASD and ADHD features in those with SYNGAP1-ID, particularly children*,* has not been explored; an important gap in the literature which we set out to address in order to help inform diagnosis and management. In this study, we therefore set out to examine three questions: (1) Do children with SYNGAP1-ID demonstrate more autistic behaviours than individuals that are typically developing? We hypothesised that children with SYNGAP1-ID will show higher levels of autistic features. (2) Do children with SYNGAP1-ID demonstrate higher levels of ADHD symptomology than typically developing children? We hypothesised that ADHD symptomology will be greater in those with SYNGAP1-ID. (3) If they do, what are the relative strengths and difficulties in regards to social and attentional behaviours that are displayed by children with SYNGAP1-ID? (4) Further, can we use cluster analysis to quantitatively examine the heterogeneity of those with SYNGAP1-ID*,* thereby providing a greater perspective and understanding into this syndrome.

## Method

### Participants

The study protocol was reviewed and approved by NHS Scotland A Research Ethics Committee. Families were recruited through patient and family organisations (SYNGAP1 Foundation and SynGAP Research Fund) and from our own research centre contact database and through our social media channels. Control participants were recruited from family members of those with SYNGAP1-ID and through our contact database. Written informed consent was obtained for all participants, either from a parent / caregiver or the participant themselves as appropriate.

### Measures

To examine autistic traits, parents and caregivers completed the Social Responsiveness Scale-2 (SRS; (Constantino & Gruber, 2012) and the Social Communication Questionnaire – Lifetime (SCQ). The Social Responsiveness Scale-2 is a 65-item parent report form which is primarily used to examine social abilities in the last six months and can be used as a screening tool and clinical aid in the diagnosis of ASD. Each item is scored on a 4-point Likert scale with total raw scores then transformed into T-scores. The higher the score, the greater the degree of social impairment with a t-score of 59 or below considered to be in the typical range; 60–65 being in the mild range; 66–75 moderate range, and 75 or greater being in the severe range. A cut-off t-score of 60 or greater results in a 96.8% likelihood of a later clinical diagnosis of ASD (Constantino et al., 2007). In addition to a total score, there are five subscales: social awareness, social cognition, social communication, social motivation, and restricted interests and repetitive behaviours (RRB). Additionally, there are two DSM-5 specific compatible scales: social communication and interaction (SCI) and restricted interests and repetitive behaviour (RRB).

The SCQ- lifetime is a 40-item, parent report measure that is a widely used screening tool for ASD for those aged 4 and older (Rutter, 2003). Each item receives a score of either 1 (indicating presence of the particular ASD-related behaviour) or 0 (indicating the absence of the behaviour) with respondents asked to indicate whether the behaviours have ever been present. The first item is not scored but determines whether the items relating to language development are required to be answered. As a result, those that are verbal are required to answer these items and as such can score a total score between 0–39. Those that are nonverbal can score a total between 0–33. A total score above 15 suggests that the individual is likely to be on the autism spectrum and would therefore warrant further assessment.

To examine ADHD behavioural manifestations, parents and caregivers completed the Conners 3—Parent questionnaire (Conners, 1997). The Conners is a widely used standardised instrument designed to assess behaviours related to ADHD in those aged between 6 and 18 years old. It consists of 11 subscales: inattention, hyperactivity/impulsivity, learning problems, executive function, defiance/aggression, peer relations, ADHD inattention, ADHD hyperactive impulsive, conduct disorder, oppositional defiant disorder, and an overall Conners global index score. It consists of 108 items, with each item score on a 4-point Likert scale. Total raw scores are then converted to t-scores, with higher scores indicating increased ADHD symptoms. The Conners has a ceiling of 90 and a floor of 40 with a mean of 50 (SD = 10). This t-score provides a developmentally sensitive measure that allows for comparison with other individuals to determine if their level of symptoms is atypical for their age, with scores above 60 indicating elevated concern. The Conners can also provide information relevant to the DSM-5 diagnosis as an absolute value, which determines whether the symptom count has been met or not. Alongside this, a probability index can also be produced, which states the probability that an individual’s score is similar to an individual who has ADHD.

The Leiter-3 International Performance scale (3^rd^ edition) was used to estimate non-verbal IQ (NVIQ). The Leiter-3 assesses cognitive functioning in individuals aged between 3 and 75 + years of age. It is a nonverbal testing instrument that has been designed to be administered to individuals with difficulties with communication. It is comprised of two batteries of subtests– cognitive and attention memory. A NVIQ score was generated from scores obtained from four subtests (Figure ground, form completion, sequential order and classification/analogies) from the cognitive battery. The raw scores from these subtests were converted to normalised scaled scores, which were summed to produce an overall NVIQ score. Normalised NVIQ had a mean of 100 (SD = 15).

### Medication Use

Information on medication use was also obtained. For the SYNGAP1-ID group, for those who provided medication use, only one individual was reported to be taking medication for ADHD (Atomoxetine). None of the typically developing controls reported being on any medication. See supplementary materials for a complete breakdown of medication use.

### Statistical Analysis

Analysis was performed on total score of the SCQ, and the t-scores for the SRS and the Conners subscales. All data was examined for normality of distribution and descriptive statistics were calculated. To compare groups on the measures, Mann–Whitney U nonparametric tests were used. Spearman’s rank order correlations were performed to examine the association between age, IQ and the scores on the separate measures. To correct for multiple comparisons a Bonferroni correction was applied for each statistical test.

To examine the heterogeneity of SYNGAP1-ID, we ran a clustering analysis to identify subgroups within this population. This approach attempts to place individuals into the same categories and thus provides empirical confirmation of clinical subtypes. It has previously been applied to groups with ASD (Elwin et al., 2017; Ruzich et al., 2016) and intellectual disabilities (Crocker et al., 2007). Firstly, the average silhouette method was used to determine optimal number of clusters. This method computes the quality of the clustering, with the optimal number of clusters (*k*) being the one that maximises the average silhouette over a range of possible *k* values (Kaufman & Rousseeuw, 2009). This was followed up with a *k*-means cluster analysis in order to classify and interpret the identified clusters. To investigate separation and stability of the clusters average silhouette width (Rousseeuw, 1987) and Jaccard coefficients (Hennig, 2007) were calculated. To examine the differences between the identified clusters, Mann–Whitney U nonparametric tests were conducted.

## Results

### Participants

In total, information was gathered about 30 children with SYNGAP1-ID (Mean age 7.5 years (SD = 3.1); 12 males) and 21 typically developing children (Mean age 8.1 years (SD = 3.1); 7 males). See Table 1 for a full breakdown of the samples characteristics.

**Table 1:** Sample characteristics for SYNGAP1-ID and typically developing controls

	SYNGAP1-ID **(N = 30)**	Typically developing controls (N = 21)
Gender (N)	12 males; 18 females	7 males; 14 females
Age (Years) mean (SD)	7.5 (SD 3.1)	8.1 (SD 3.1)
ASD diagnosis (N)	12 (60%)	0
ADHD diagnosis (N)	2 (7%)	0
Epilepsy diagnosis (N)	18 (60%)	0
Non-verbal IQ; mean (SD)	62 (SD 15)	107 (SD 14)

An NVIQ score was only obtained from 30 children (SYNGAP1-ID 20; Typically developing children 10), who were able to travel to the research site. There was a significant difference between those with SYNGAP1-ID and those typically developing for NVIQ (U = 2, p < 0.001). There was found to be no significant difference in age between the two groups.

### Autistic Traits (Current): SRS Cut-offs

We first looked at the scores on the SRS between the SYNGAP1-ID group and the typically developing control group. The SYNGAP1-ID group had a higher total score (Mean = 78.6, SD = 10.5) on the SRS than the typically developing controls (Mean = 43.1, SD = 4.1). For the SYNGAP1-ID group, the majority of children (N = 29; 97%) scored above the clinical cut off t-score of 60 on the total score, with 13% (N = 4) in the mild, 16% (N = 5) in the moderate and another 73% (N = 20) in the severe range. Of those who scored in the severe range, seven had already received an ASD diagnosis. For the typically developing control group, all children scored within typical limits for total score. This difference in total score between the SYNGAP1-ID group and the typically developing controls was found to be statistically significant (U = 0, p < 0.001).

**Fig. 1:**
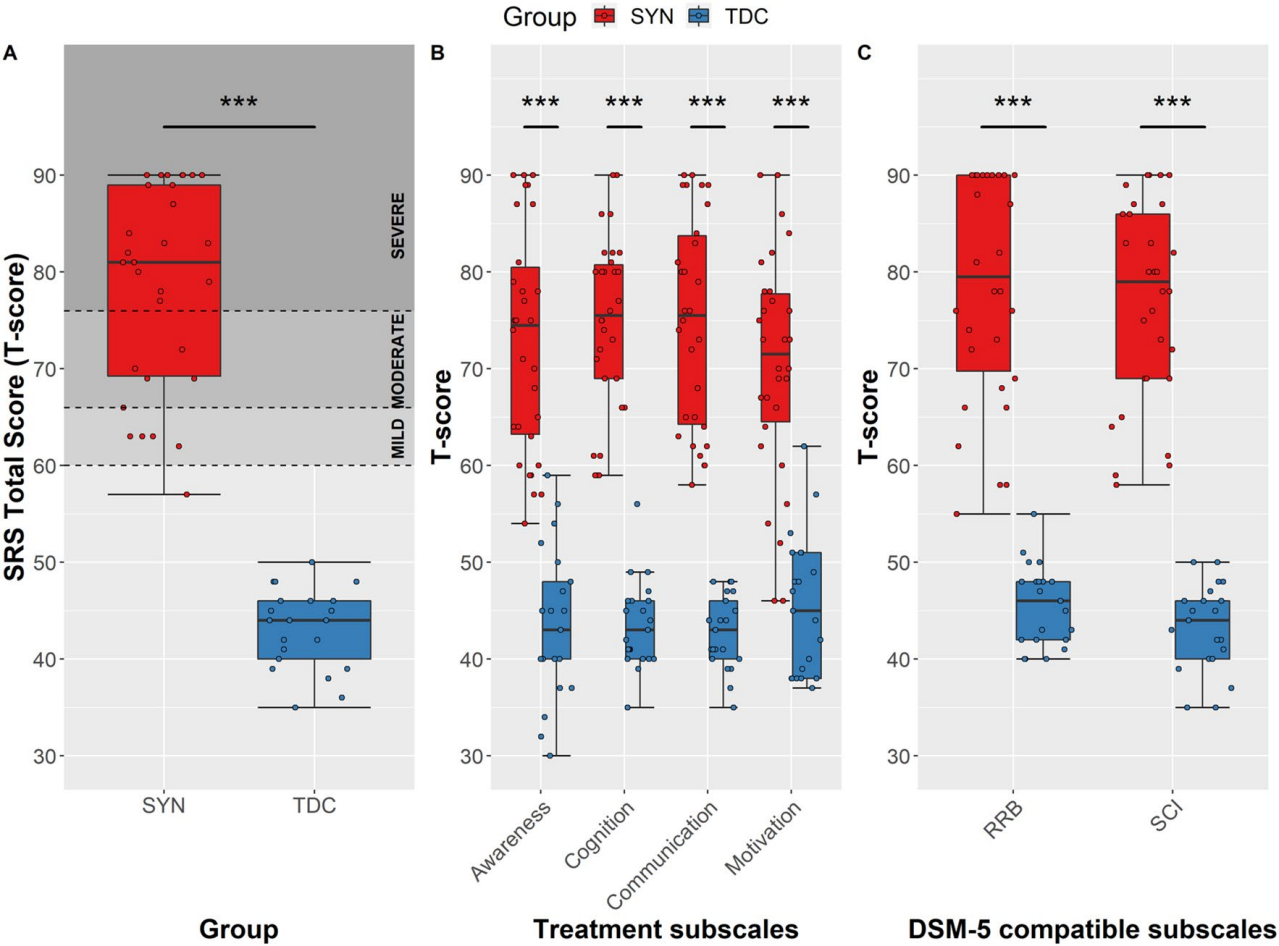
Parent reported scores on the Social Responsiveness Scale (SRS). A) SRS Total score for SYNGAP1-ID (SYN) and typically developing controls (TDC). Clinical cut-offs indicated by dotted line (Typical range ≤ 59; Mild 60–65; Moderate 66–75; and Severe ≥ 76). B) T-scale scores for SRS subscales for SYNGAP1-ID and typically developing controls. C) T-scale scores for DSM-5 compatible SRS subscales for SYNGAP1-ID and typically developing controls. Boxes correspond to interquartile range (25^th^ to 75^th^), with the minimum/maximum whiskers calculated as Q1/Q3 −/+ 1.5 times IQR. ***p ≤ 0.001

Next, we examined the SRS subscales. Children in the SYNGAP1-ID group scored highest for restricted interests and repetitive behaviours (mean = 78.5; SD = 11.6), with 63% (N = 19) in the severe and 23% (N = 7) in moderate ranges; whilst they scored lowest for social motivation (Mean = 70.3, SD = 11.7), with 16% (N = 5) in the normal range. For all the subscales, the typically developing control group scored within typical limits (t-score < 60), except one child who scored above the clinical cut-off for motivation. For these subscales, the SYNGAP1-ID group scored higher on all of the subscales compared to the typically developing controls (Fig. 1B and 1C: Awareness U = 5.5, p < 0.001; Cognition U = 0, p < 0.001; Communication U = 0, p < 0.001; Motivation U = 28.5, p < 0.001; DSM-5 RRB U = 0.5, p < 0.001; DSM-5 SCI U = 0, p < 0.001).

There were no significant differences in scores on the SRS between those in the SYNGAP1-ID group that had received an ASD diagnosis and those that had not. Although they did not survive Bonferroni correction, statistical analysis highlighted that there was a significant difference for cognition (U = 30.5, p = 0.014), motivation (U = 39.5, p = 0.044), SCI (U = 37.5, p = 0.035) between those that were verbal and nonverbal in the SYNGAP1-ID group. There were no differences between SYNGAP1-ID males and SYNGAP1-ID females on any of SRS scales.

### Autistic Traits (Current): SRS Correlations with Age and NVIQ

For the SYNGAP1-ID group, there was a significant negative correlation between NVIQ and SRS total score (r(20) = -0.541, p = 0.014), indicating that as NVIQ decreased, SRS total score increased. There was no significant correlation between age and SRS total score. For the typically developing group, there were no significant correlations between these variables. For the SYNGAP1-ID group, although they did not survive Bonferroni correction, there was a negative correlation between NVIQ and awareness (r(20) = -0.490, p = 0.028) and between NVIQ and cognition (r(20) = -0.492, p = 0.028). There was also a significant negative correlation between NVIQ and DSM-5 SCI (r(20) = -0.628, p = 0.003), which did survive the multiple comparisons correction. These correlations suggest that for those with SYNGAP1-ID as NVIQ decreases, levels of autistic traits increase. In regards to the effect of age on social behaviours there was found to be no significant correlations with any of the SRS scales. For the typically developing controls they showed no significant correlations between age and any of the SRS subscales. For NVIQ and the SRS subscales, there were no significant correlations.

### Autistic Traits (Lifetime): SCQ

**Fig. 2:**
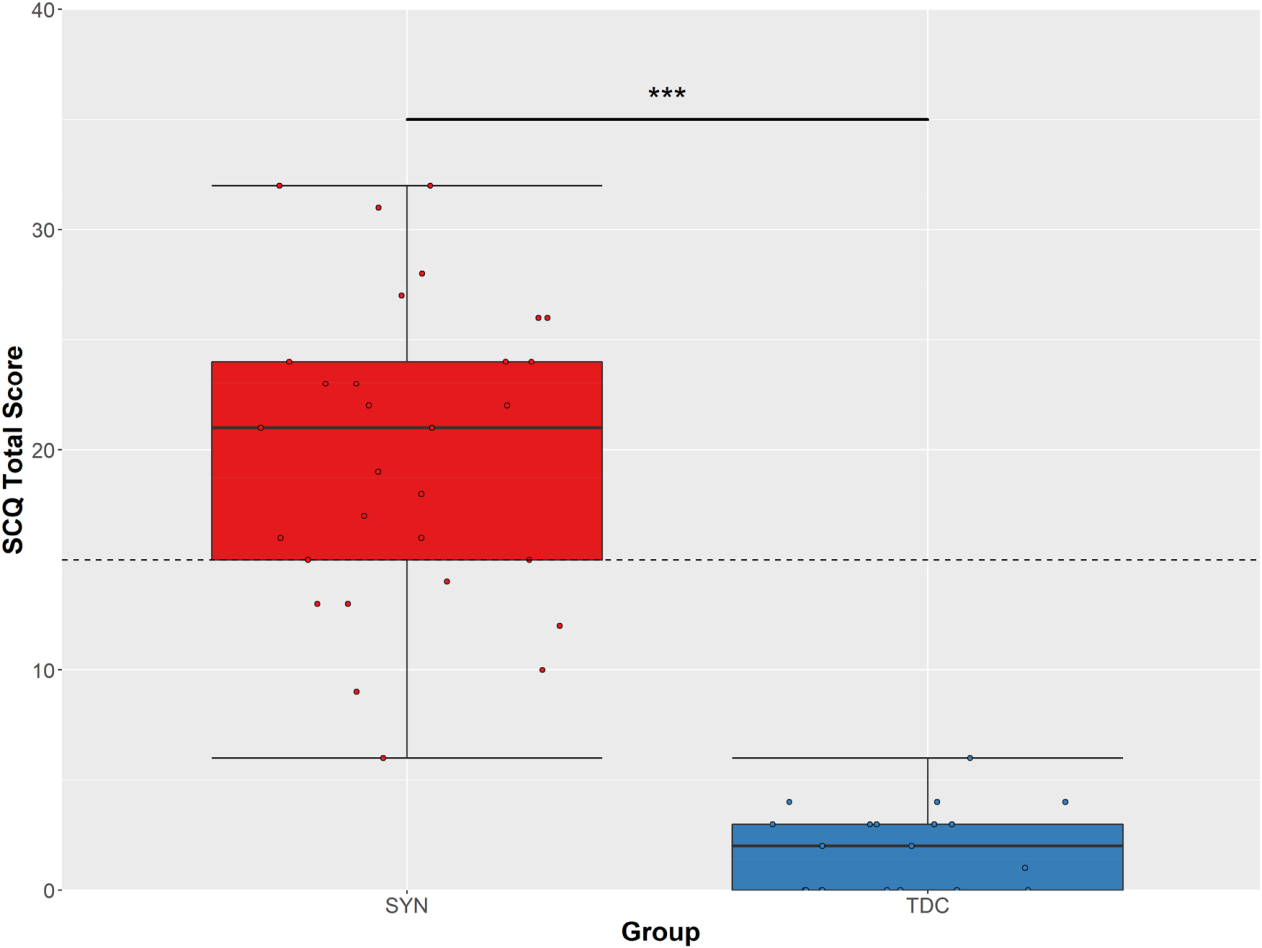
Parent reported scores on the Social Communication Questionnaire (SCQ). SCQ total score for SYNGAP1-ID (SYN) and typically developing controls (TDC). Clinical cut-off (≥ 15) indicated by dotted line. Boxes correspond to interquartile range (25^th^ to 75^th^), with the minimum/maximum whiskers calculated as Q1/Q3 −/+ 1.5 times IQR. ***p ≤ 0.001

Children with SYNGAP1-ID had higher total scores on the SCQ than typically developing controls (SYNGAP1-ID mean = 19.9, SD = 6.9; Typically developing control mean = 1.9, SD = 1.8; Fig. 2) and there was a statistically significant difference (U = 0.5, p < 0.001) between the two groups. Twenty-one (70%) children with SYNGAP1-ID scored above the cut-off of 15, indicating a positive screening for ASD, whilst of the 12 who had received a formal ASD diagnosis, 11 scored above the cut-off. For the SYNGAP1-ID children, there were no significant differences in total SCQ score between males and females or between those that had received an ASD diagnosis and those that had not. There were no significant differences in total SCQ score between those with SYNGAP1-ID that were verbal and non-verbal. None of the typically developing control group scored above the cut-off.

### Autistic Traits (Lifetime): SCQ Correlations

For the SYNGAP1-ID group, there was no significant correlation between age and total SCQ score. There was also a significant negative correlation (r(20) -0.789; p < 0.001) between NVIQ and total score on the SCQ. For the typically developing control group, there was no significant correlations between either IQ or age and total score on the SCQ.

### ADHD Traits: Conners 3 Parent

A subset of children (n = 19) aged between 6–18 years also completed the Conners-3 parent scale. For all subscales on the Conners, the SYNGAP1-ID group scored higher than the typically developing controls. For the ADHD probability index, which indicates whether the individual is similar to an individual with ADHD, most (n = 16/19; 84%) of the SYNGAP1-ID group were found to have a ADHD probability Index of greater than 50%, whilst for the typically developing controls only two individuals out of 17 were found to have a score above this percentage. On the Conners Global Index, which is an indicator of overall psychopathology, most of the SYNGAP1-ID group (N = 17/19; 89%) scored above the clinical cut-off of 60.

**Fig. 3:**
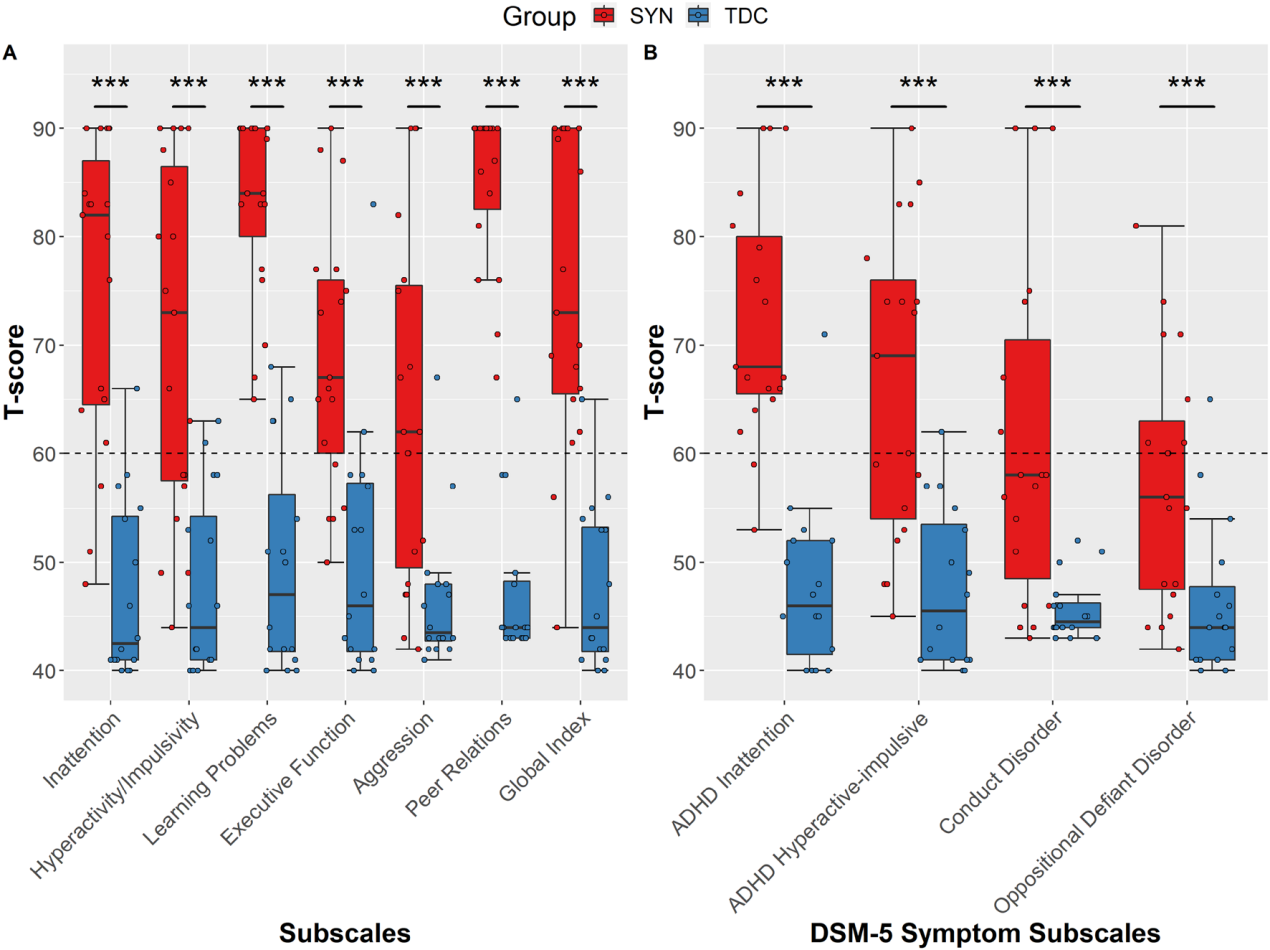
Parent reported Conners scores for SYNGAP1-ID (SYN) and typically developing controls (TDC). A) Scores on the Conners subscales. B) Scores on Conners DSM-5 orientated scales. Clinical cut-offs indicated by dotted line (≥ 60). Boxes correspond to interquartile range (25^th^ to 75^th^), with the minimum/maximum whiskers calculated as Q1/Q3 −/+ 1.5 times IQR. ***p ≤ 0.001

Scores for the SYNGAP1-ID group demonstrated that they had particular difficulties with peer relations (Mean t-score 85.1; SD 7.3), with 18 of those having a t-score in the very elevated range (> 70; 98^th^ percentile). The SYNGAP1-ID displayed low scores in aggression (Mean t-score = 63.79; SD = 16.25) suggesting this area as a strength, with 37% (N = 7) children in the typical level of concern range. After Bonferroni correction, significant differences were found across the subscales between the SYNGAP1-ID group and the typically developing controls (Inattention U = 17, p < 0.001; Hyperactivity U = 34.5, p < 0.001; Learning problems U = 2.5, p < 0.001; Executive function U = 36, p =  < 0.001; Aggression U = 47, p < 0.001; Peer relations U = 0, p < 0.001; Global Index U = 12, p < 0.001) (Fig. 3A).

For the DSM-5 subscales, scores indicated that for those with SYNGAP1-ID there was a relatively low occurrence of symptoms for DSM-5 conduct disorder and DSM-5 oppositional defiant disorder, with 12 (63%) children with SYNGAP1-ID scoring within typical levels of concern. Those with SYNGAP1-ID had particular weakness in ADHD inattention (Mean 72.05; SD 10.9), with 79% (n = 15) scoring in the elevated or very elevated t-score range (t-score ≥ 65). There was found to be significant differences between the SYNGAP1-ID group and the typically developing controls on all four DSM-5 subscales (Inattention U = 12.5, p < 0.001; Hyperactivity U = 38, p < 0.001; Conduct disorder U = 47, p < 0.001; Oppositional defiant disorder U = 57 p = 0.001) (Fig. 3B).

In regards to those with SYNGAP1-ID, there were no significant differences in Conners t-scale scores between those with an ASD diagnosis and those without. There were also no significant differences between those with SYNGAP1-ID that were reported as being verbal and those SYNGAP1-ID individuals that were non-verbal. In regards to the effect of gender on scores from the Conners, there was found to be a single significant difference for learning problems (U = 12, p = 0.006) between SYNGAP1-ID males and SYNGAP1-ID females, although it did not survive multiple comparisons corrections.

Alongside the relative t-score, the Conners also produces an absolute score, which determines whether or not an individual displays symptoms of a particular DSM-5 diagnostic criteria. For those with SYNGAP1-ID, 42% (N = 8) displayed symptoms of ADHD inattention, 32% (N = 6) for hyperactivity, 37% (N = 7) for conduct disorder and 21% (N = 4) showed oppositional defiant disorder symptoms. None of the typically-developing controls met the symptom criteria on any of the DSM-5 subscales.

### ADHD Traits: Conners 3 Parent Correlations

Next, correlations were run to examine whether there were any associations between the scores on the Conners with either age or NVIQ. For the SYNGAP1-ID group, no significant associations were uncovered between age and scores on the subscales. For NVIQ, there was a significant negative correlation only for peer relations (r(14) = − 0.598, p = 0.024) for those with SYNGAP1-ID*,* however this was not present after Bonferroni correction. For the typically developing controls, there were no associations between NVIQ and the scales on the Conners. Nevertheless, for age there was a significant negative correlation with Conduct Disorder (r(16) = − 0.507, p = 0.045), although this did not survive multiple comparisons correction.

### Clustering and Heterogeneity of SYNGAP1-ID

As expected our SYNGAP1-ID group scored highly for ASD and ADHD traits compared with the typically developing controls. However, within those with SYNGAP1-ID and across the measures we observed a wide distribution of scores, with some individuals scoring highly, whilst others presented a milder social and attentional impairment profile. To explore this SYNGAP1-ID heterogeneity and to identify subgroups, we performed separate cluster analyses focusing on autism and ADHD traits. To assess autism traits, scores from the SRS subscales (Awareness, cognition, communication, motivation, and RRB) were combined into a single cluster analysis. The SCI was not included as this subscale is calculated from the summation of the other subscales. To assess ADHD, the Conners subscales (Inattention, hyperactivity, learning problems, executive function, defiance/aggression, and peer relations) were examined with a second cluster analysis.

### Autism Traits

**Fig. 4:**
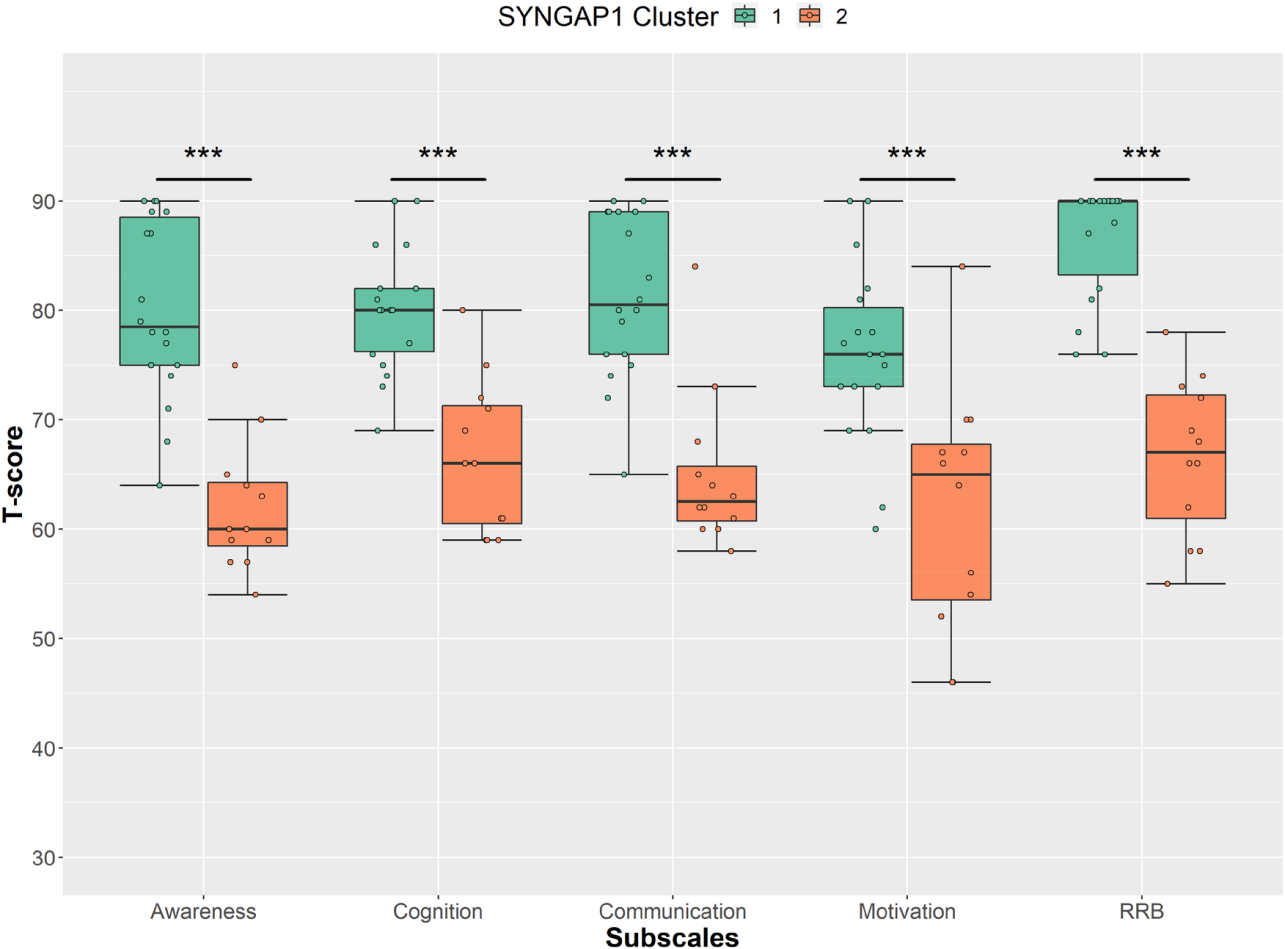
Parent reported scores on the Social Responsiveness Scale subscales for those with SYNGAP1-ID grouped by cluster. A) T-scores on the SRS subscales (Awareness, cognition, communication, motivation and RRB) for those with SYNGAP1-ID grouped by cluster. Boxes correspond to interquartile range (25^th^ to 75^th^), with the minimum/maximum whiskers calculated as Q1/Q3 -/ + 1.5 times IQR.. *** p ≤ 0.001

For social impairments, cluster analysis identified two clusters. Cluster 1 contained 18 children whilst cluster 2 contained 12 children (Table 2). For this cluster analysis solution, silhouette width was calculated as 0.46 and the Jaccard coefficient was > 0.85 indicating that the clusters were well separated and stable. Cluster 1 scored significantly higher for all the SRS subscales (Awareness U = 8.5, p < 0.001; Cognition U = 14, p < 0.001; Communication U = 14.5, p < 0.001; Motivation U = 31, p < 0.001; RRB U = 2.5, p < 0.001) than cluster 2 (Fig. 4). There was no difference in age between the two clusters, but there was a difference in NVIQ (U = 18.5, p = 0.017), although it did not survive multiple comparisons correction.

**Table 2:** Demographics of each SYNGAP1-ID cluster for social and attentional impairments

	ASD traits		ADHD traits	
	Cluster 1	Cluster 2	Cluster 1	Cluster 2
N	18	12	9	10
Age (Mean)	7.8 years (SD 3.5)	6.9 years (SD 2.3)	8.4 years (SD 0.8)	10.1 years (SD 2.8)
Gender (N)	7 Males; 11 Females	5 Males; 7 Females	5 Male; 4 Females	3 Males; 7 Females
NVIQ (Mean)	57 (SD 9)	68 (SD 18)	65 (SD 15)	55 (SD 11)
ASD diagnosis (N)	8	4	5	5
Epilepsy diagnosis (N)	11	7	3	8

### ADHD Traits

**Fig. 5:**
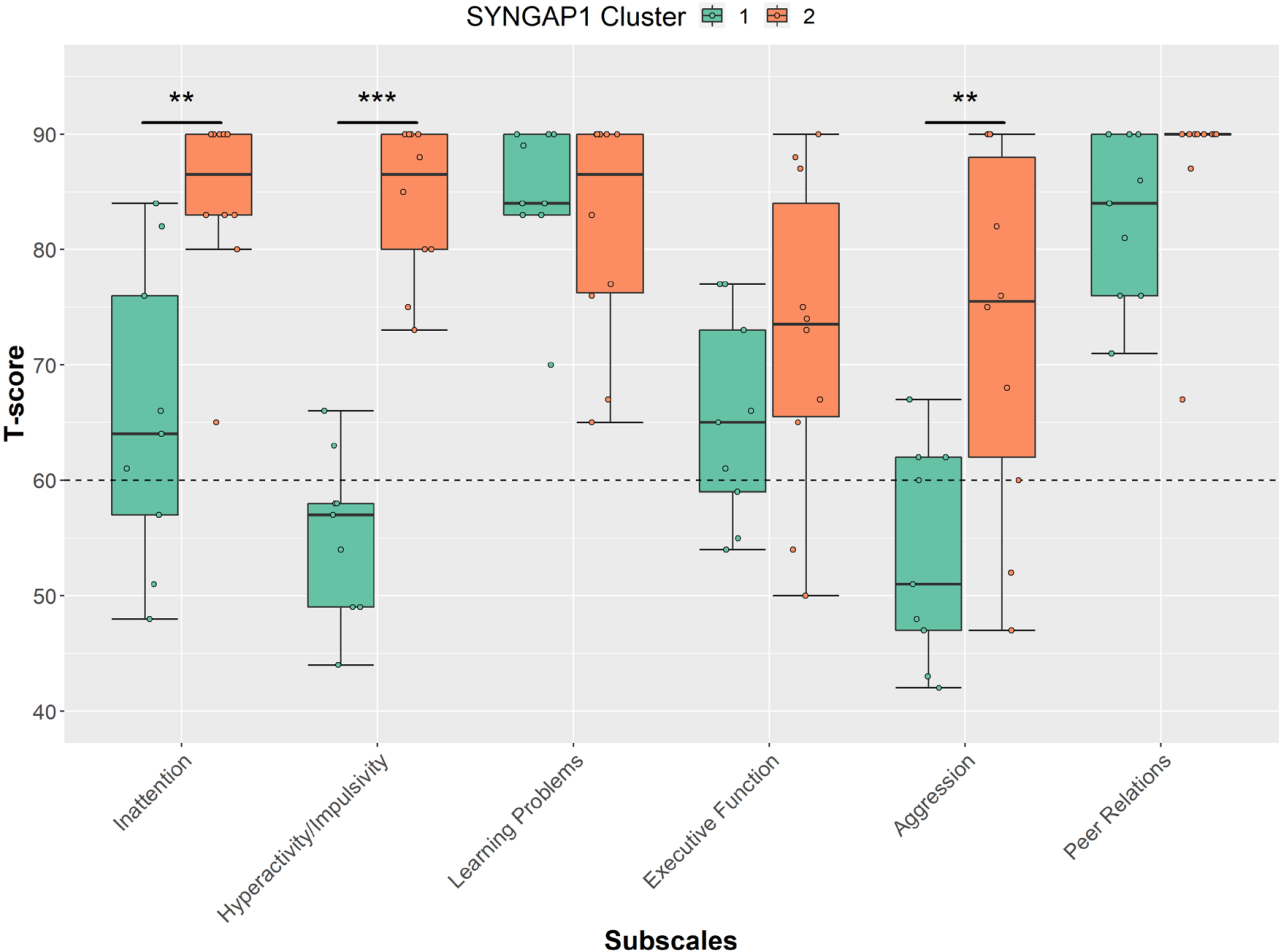
Parent reported scores on the Conners subscales for those with SYNGAP1-ID grouped by cluster. Clinical cut-offs indicated by dotted line (≥ 60). Boxes correspond to interquartile range (25^th^ to 75^th^), with the minimum/maximum whiskers calculated as Q1/Q3 -/ + 1.5 times IQR. *** p ≤ 0.001; ** p ≤ 0.01

Based on ADHD traits, analysis identified that the SYNGAP1-ID group contained two clusters, with cluster 1 containing 9 children and cluster 2 containing 10 (Table 2). Silhouette width for this clustering solution was calculated as 0.36 with the Jaccard coefficient as > 0.85. Cluster 1 was found to score significantly lower on inattention (U = 9, p = 0.002), and hyperactivity (U = 0, p < 0.001). Those in cluster 1 also scored significantly lower on aggression (U = 14, p = 0.01) than cluster 2, although this did not survive multiple comparisons correction (Fig. 5). There was found to be no differences in age or NVIQ between the two clusters.

As the cluster analysis had identified that for both ASD and ADHD traits, there were two clusters, one which scored highly and a second cluster with generally lower scores we were interested in how these were associated with one another (Table 3). We found that there was a significant association between the clusters *X*^2^(1) = 4.232, p = 0.04), with those scoring high for ASD likely to score highly for ADHD traits.

**Table 3:** Cross tabulation of each SYNGAP1-ID cluster scoring high and low for social and attentional impairments

		ADHD traits		
		High scorer (N)	Low scorer (N)	Total (N)
ASD traits	High scorer (N)	8	3	11
	Low scorer (N)	2	6	8
	Total (N)	10	9	19

## Discussion

Presently, little is known of the clinical presentation and aetiology of the social impairments and attentional differences of children with SYNGAP1-ID. Previous findings have highlighted that the prevalence of ASD in SYNGAP1-ID ranges from 50–73%, whilst little research has so far been conducted to examine the profile of ADHD in this population. Through the use of parent-completed questionnaires we attempted to shed light on these. We found that high levels of autistic and ADHD traits were seen in children with SYNGAP1-ID, including many who score above the standardised cut-offs for the scales used. Within the SYNGAP1-ID group autistic traits in particular were associated with a lower non-verbal IQ, whereas ADHD traits were generally not. Neither autistic nor ADHD traits were associated with age.

Those with SYNGAP1-ID demonstrated high levels of social impairment compared to the typically developing controls. As ASD is thought to be highly prevalent amongst the SYNGAP1-ID population, we were unsurprised to find children scoring highly on both of the ASD measures (Mignot et al., 2016). For the SRS, 97% of SYNGAP1-ID children were reported to have a total SRS t-score greater than 60, scoring highest for restricted interests and repetitive behaviours, and lowest for social motivation. For the SCQ, 70% of children with SYNGAP1-ID scored above the cut-off of 15, indicating a positive screening for ASD. This is greater than previously reported by some SYNGAP1-ID studies (e.g. Mignot et al., 2016). In practice, only 44% of our SYNGAP1-ID group had received a formal ASD diagnosis. On one hand this may indicate an area of unmet need, given that individuals could benefit from extra or more tailored support or intervention, for example in educational or care/support settings. On the other hand, previous studies have suggested that the SRS in particular may have limited diagnostic utility in populations with significant intellectual disability (Gergoudis et al., 2020). Consistent with this, both SRS and SCQ scores in our sample correlated negatively with non-verbal IQ, i.e. those with lower IQ displayed higher scores on these scales, suggesting that at least some of the identified traits may relate to global intellectual impairment. Overall, we would suggest that while autism should be considered as a potential additional diagnosis for all individuals with SYNGAP1-ID, careful and detailed clinical assessment will be required to determine whether the displayed autistic traits are greater than would be expected given an individual’s developmental level.

Little is known about the presentation of ADHD in individuals with intellectual disabilities and this is the first study to quantify attentional dysfunction and broader ADHD traits in SYNGAP1-ID. Within the SYNGAP1-ID group there was a high prevalence of ADHD traits, with the majority (84%) of the group scoring either a high or very high probability on the ADHD Index. This is in line with other genetic syndromes. For example, using the Conners rating scale, Newman et al. (2015) found that problems with attention and hyperactivity in fragile X syndrome were highly prevalent, with 83% of participants demonstrating ADHD symptoms. The high prevalence of ADHD symptoms seen for our SYNGAP1-ID group suggests that currently ADHD is underdiagnosed as only 6% of our SYNGAP1-ID group had received a formal diagnosis of ADHD. Of particular importance is that, with the exception of the peer relations subscale, the Conners scores were not significantly associated with NVIQ, suggesting that they are not the result of global developmental delay and highlighting the importance of their identification as potentially independent features.

Although some trends were observed, we found that there were no significant relationships between age and scores on either the SRS, SCQ, or the Conners for those with SYNGAP1-ID. Changes in autistic traits over time would not necessarily be expected, but ADHD traits are generally thought to moderate somewhat over time in the general population. Our findings suggest that this may not be the case in those with SYNGAP1-ID. However, it is important to be aware that this lack of correlation may have been the result of the distribution of ages within this sample, with the SYNGAP1-ID group being mainly of school age. Further the cross-sectional design of this study means that any age relationships identified must be confirmed in longitudinal studies. Future research should examine more closely the relationship between age and social and attentional impairments to cover a greater age range with a larger sample size at multiple time points across development.

Despite the high prevalence of ASD and ADHD traits amongst those with SYNGAP1-ID, scores across the measures demonstrated a high variability with some individuals exhibiting fewer traits, similar to some typically developing controls whilst others were scoring at ceiling on the rating scales. This heterogeneity could pose a challenge for clinical definitions of SYNGAP1-ID. As such, we explored the heterogeneity in SYNGAP1-ID in order to improve phenotypic homogeneity. We found that both social and attentional impairments could each be grouped into two clusters: one which scored highly and a second cluster with generally lower scores. This is the first study to examine heterogeneity and subtypes with SYNGAP1-ID. Within the ASD literature, previous studies have tried to tease out the degree to which ASD could be sub-classified dimensionally or categorically, i.e. whether ‘distinct subtypes’ (e.g. (Eaves et al., 1994) existed or whether a ‘severity gradient’ (Ring et al., 2008) was a more appropriate way to consider heterogeneity. In their review of the literature, Syriopoulou-Delli & Papaefstathiou (2020) concluded that drawing conclusions on this dichotomy was difficult and that using both classifications may be necessary to fully understand heterogeneity. It has been suggested that presence/absence and degree of co-occurring intellectual disability may be used as a basis to distinguish ASD subgroups (Mayes & Calhoun, 2011; Munson et al., 2008) with Munson et al. (2008) finding that for ASD subtypes, higher overall cognitive ability was associated with lower levels of autistic traits. It is important to note however, that level of adaptive functioning or degree of distress does not necessarily map linearly to either level of autistic traits or cognitive ability. Interestingly, in our cluster analysis, NVIQ did not differ between SYNGAP1-ID clusters on either ASD or ADHD traits, although within the full SYNGAP1-ID group higher levels of ASD traits were correlated with lower NVIQ, a pattern not seen in regards to ADHD traits. Alongside this, there were no differences in age between the clusters. Together, these findings add to our understanding of the complexity of presentations in SYNGAP1-ID and that distinct groupings, independent of overall cognitive ability may be able to be identified. Future research should look to further establish the possible causes for the variation in the SYNGAP1-ID behavioural profile.

These findings should be considered in light of a number of limitations. Firstly, the behavioural characteristics reported here were collected via informant completed measures. As such the findings may be subject to measurement bias and error variance and therefore only provide a summary judgment of the behaviours rather than being a direct measure. Secondly, our sample size may limit the reliability of the cluster analysis performed, with larger samples potentially representing a broader range of ages and clinical presentations. In particular, Dalmaijer et al. (2022) recommended an N of at least 20 per subgroup in order to have sufficient statistical power, although the cluster separation and stability scores suggest that the analysis maintains validity despite the relatively low numbers. Our findings would also have benefitted from the inclusion of a non-SYNGAP1-ID group to provide more information about how the ASD and ADHD symptomatology of SYNGAP1-ID compares with others with ID. Finally, as noted above, the cross-sectional design of the study limits the degree to which conclusions can be drawn about age relationships; longitudinal studies are required to confirm or refute these.

In conclusion, we set out to uncover if there was a specific endophenotype of ASD and ADHD for children with SYNGAP1-ID*.* We found that those with SYNGAP1-ID demonstrated high levels of both social impairments and attentional deficits, and particularly high levels of restricted interests and repetitive behaviours along with difficulties with peer relations. However, they demonstrated strengths in their awareness of social cues and were reported to have low levels of conduct disorder and oppositional defiant disorder symptoms. These findings indicate that the majority of children with SYNGAP1-ID should be assessed for ASD and ADHD, although traits of ASD may relate more to global intellectual impairment. Scores across all three measures were found to have a high variance, with some children scoring at ceiling whilst others were judged to be at around the clinical cut-off. Hierarchical cluster analysis identified that the SYNGAP1-ID group could be clustered into low and high scoring on both ASD and ADHD traits. These findings shed new light on the profile of SYNGAP1-ID and have important implications in helping to establish effective interventions for those with SYNGAP1-ID and to improve access to resources for families.

## Supplementary Information

Below is the link to the electronic supplementary material.

Supplementary file1 (DOCX 22 KB)

## Data Availability

The datasets for study are available from the corresponding author on reasonable request.

## References

[CR1] Abrahams, B. S., & Geschwind, D. H. (2008). Advances in autism genetics: On the threshold of a new neurobiology. *Nature Reviews Genetics,**9*(5), 5. 10.1038/nrg2346 10.1038/nrg2346 PMC2756414 18414403

[CR2] Conners, C. K. (1997). *Conners' parent rating scales: Revised*. Multi-Health Systems.

[CR3] Constantino, J. N., & Guber, C. P. (2012). *Social Responsiveness Scale Second Edition (SRS-2): Manual*. Western Psychological Services.

[CR4] Constantino, J. N., Lavesser, P. D., Zhang, Y., Abbacchi, A. M., Gray, T., & Todd, R. D. (2007). Rapid Quantitative Assessment of Autistic Social Impairment by Classroom Teachers. *Journal of the American Academy of Child & Adolescent Psychiatry,**46*(12), 1668–1676. 10.1097/chi.0b013e318157cb23 18030089 10.1097/chi.0b013e318157cb23

[CR5] Crocker, A. G., Mercier, C., Allaire, J.-F., & Roy, M.-E. (2007). Profiles and correlates of aggressive behaviour among adults with intellectual disabilities. *Journal of Intellectual Disability Research,**51*(10), 786–801. 10.1111/j.1365-2788.2007.00953.x 17803497 10.1111/j.1365-2788.2007.00953.x

[CR6] Dalmaijer, E. S., Nord, C. L., & Astle, D. E. (2022). Statistical power for cluster analysis. *BMC Bioinformatics,**23*(1), 205. 10.1186/s12859-022-04675-1 35641905 10.1186/s12859-022-04675-1 PMC9158113

[CR7] Dekker, M. C., & Koot, H. M. (2003). DSM-IV Disorders in Children With Borderline to Moderate Intellectual Disability. I: Prevalence and Impact. *Journal of the American Academy of Child & Adolescent Psychiatry,**42*(8), 915–922. 10.1097/01.CHI.0000046892.27264.1A 12874493 10.1097/01.CHI.0000046892.27264.1A

[CR8] Eaves, L. C., Ho, H. H., & Eaves, D. M. (1994). Subtypes of autism by cluster analysis. *Journal of Autism and Developmental Disorders,**24*(1), 3–22. 10.1007/BF02172209 8188572 10.1007/BF02172209

[CR9] Elwin, M., Schröder, A., Ek, L., Wallsten, T., & Kjellin, L. (2017). Sensory Clusters of Adults With and Without Autism Spectrum Conditions. *Journal of Autism and Developmental Disorders,**47*(3), 579–589. 10.1007/s10803-016-2976-1 27921201 10.1007/s10803-016-2976-1 PMC5352790

[CR10] Gergoudis, K., Weinberg, A., Templin, J., Farmer, C., Durkin, A., Weissman, J., Siper, P., Foss-Feig, J., del Pilar Trelles, M., Bernstein, J. A., Buxbaum, J. D., Berry-Kravis, E., Powell, C. M., Sahin, M., Soorya, L., Thurm, A., & Kolevzon, A. (2020). Psychometric Study of the Social Responsiveness Scale in Phelan–McDermid Syndrome. *Autism Research : Official Journal of the International Society for Autism Research,**13*(8), 1383–1396. 10.1002/aur.2299 32406614 10.1002/aur.2299 PMC8103889

[CR11] Harpin, V., Mazzone, L., Raynaud, J. P., Kahle, J., & Hodgkins, P. (2016). Long-Term Outcomes of ADHD: A Systematic Review of Self-Esteem and Social Function. *Journal of Attention Disorders,**20*(4), 295–305. 10.1177/1087054713486516 23698916 10.1177/1087054713486516

[CR12] Hennig, C. (2007). Cluster-wise assessment of cluster stability. *Computational Statistics & Data Analysis,**52*(1), 258–271. 10.1016/j.csda.2006.11.025

[CR13] Hirota, T., & King, B. H. (2023). Autism Spectrum Disorder: A Review. *JAMA,**329*(2), 157–168. 10.1001/jama.2022.23661 36625807 10.1001/jama.2022.23661

[CR14] Holder, J. L., Hamdan, F. F., & Michaud, J. L. (2019). SYNGAP1-Related Intellectual Disability. In *GeneReviews®[Internet]*. Seattle: University of Washington. 30789692

[CR15] Jimenez-Gomez, A., Niu, S., Andujar-Perez, F., McQuade, E. A., Balasa, A., Huss, D., Coorg, R., Quach, M., Vinson, S., Risen, S., & Holder, J. L. (2019). Phenotypic characterization of individuals with SYNGAP1 pathogenic variants reveals a potential correlation between posterior dominant rhythm and developmental progression. *Journal of Neurodevelopmental Disorders,**11*(1), 18. 10.1186/s11689-019-9276-y 31395010 10.1186/s11689-019-9276-y PMC6688356

[CR16] Kaufman, L., & Rousseeuw, P. J. (2009). *Finding Groups in Data: An Introduction to Cluster Analysis*. Hoboken: Wiley.

[CR17] La Malfa, G., Lassi, S., Bertelli, M., Salvini, R., & Placidi, G. F. (2004). Autism and intellectual disability: A study of prevalence on a sample of the Italian population. *Journal of Intellectual Disability Research,**48*(3), 262–267. 10.1111/j.1365-2788.2003.00567.x 15025669 10.1111/j.1365-2788.2003.00567.x

[CR18] Lesniak-Karpiak, K., Mazzocco, M. M. M., & Ross, J. L. (2003). Behavioral Assessment of Social Anxiety in Females with Turner or Fragile X Syndrome. *Journal of Autism and Developmental Disorders,**33*(1), 55–67. 10.1023/A:1022230504787 12708580 10.1023/a:1022230504787

[CR19] Lo-Castro, A., D’Agati, E., & Curatolo, P. (2011). ADHD and genetic syndromes. *Brain and Development,**33*(6), 456–461. 10.1016/j.braindev.2010.05.011 20573461 10.1016/j.braindev.2010.05.011

[CR20] Mayes, S. D., & Calhoun, S. L. (2011). Impact of IQ, age, SES, gender, and race on autistic symptoms. *Research in Autism Spectrum Disorders,**5*(2), 749–757. 10.1016/j.rasd.2010.09.002

[CR21] Mignot, C., von Stülpnagel, C., Nava, C., Ville, D., Sanlaville, D., Lesca, G., Rastetter, A., Gachet, B., Marie, Y., Korenke, G. C., Borggraefe, I., Hoffmann-Zacharska, D., Szczepanik, E., Rudzka-Dybała, M., Yiş, U., Çağlayan, H., Isapof, A., Marey, I., Panagiotakaki, E., & Depienne, C. (2016). Genetic and neurodevelopmental spectrum of SYNGAP1-associated intellectual disability and epilepsy. *Journal of Medical Genetics,**53*(8), 511–522. 10.1136/jmedgenet-2015-103451 26989088 10.1136/jmedgenet-2015-103451

[CR22] Moss, J., & Howlin, P. (2009). Autism spectrum disorders in genetic syndromes: Implications for diagnosis, intervention and understanding the wider autism spectrum disorder population. *Journal of Intellectual Disability Research,**53*(10), 852–873. 10.1111/j.1365-2788.2009.01197.x 19708861 10.1111/j.1365-2788.2009.01197.x

[CR23] Moss, J., Oliver, C., Nelson, L., Richards, C., & Hall, S. (2013). Delineating the Profile of Autism Spectrum Disorder Characteristics in Cornelia de Lange and Fragile X Syndromes. *American Journal on Intellectual and Developmental Disabilities,**118*(1), 55–73. 10.1352/1944-7558-118.1.55 23301903 10.1352/1944-7558-118.1.55

[CR24] Munson, J., Dawson, G., Sterling, L., Beauchaine, T., Zhou, A., Koehler, E., Lord, C., Rogers, S., Sigman, M., Estes, A., & Abbott, R. (2008). Evidence for Latent Classes of IQ in Young Children With Autism Spectrum Disorder. *American Journal on Mental Retardation,**113*(6), 439–452. 10.1352/2008.113:439-452 19127655 10.1352/2008.113:439-452 PMC2991056

[CR25] Newman, I., Leader, G., Chen, J. L., & Mannion, A. (2015). An analysis of challenging behavior, comorbid psychopathology, and Attention-Deficit/Hyperactivity Disorder in Fragile X Syndrome. *Research in Developmental Disabilities, **38*, 7–17. 25543996 10.1016/j.ridd.2014.11.003

[CR26] Richards, C., Jones, C., Groves, L., Moss, J., & Oliver, C. (2015). Prevalence of autism spectrum disorder phenomenology in genetic disorders: A systematic review and meta-analysis. *The Lancet Psychiatry,**2*(10), 909–916. 10.1016/S2215-0366(15)00376-4 26341300 10.1016/S2215-0366(15)00376-4

[CR27] Ring, H., Woodbury-Smith, M., Watson, P., Wheelwright, S., & Baron-Cohen, S. (2008). Clinical heterogeneity among people with high functioning autism spectrum conditions: Evidence favouring a continuous severity gradient. *Behavioral and Brain Functions,**4*(1), 11. 10.1186/1744-9081-4-11 18289376 10.1186/1744-9081-4-11 PMC2265729

[CR28] Rousseeuw, P. J. (1987). Silhouettes: A graphical aid to the interpretation and validation of cluster analysis. *Journal of Computational and Applied Mathematics,**20*, 53–65. 10.1016/0377-0427(87)90125-7

[CR29] Rutter, M. (2003). Rutter M, Bailey A & Lord C (2003). The Social Communication Questionnaire. Los Angeles: Western Psychological Services.

[CR30] Ruzich, E., Allison, C., Smith, P., Watson, P., Auyeung, B., Ring, H., & Baron-Cohen, S. (2016). Subgrouping siblings of people with autism: Identifying the broader autism phenotype. *Autism Research,**9*(6), 658–665. 10.1002/aur.1544 26332889 10.1002/aur.1544 PMC4915503

[CR31] Sacco, R., Camilleri, N., Eberhardt, J., Umla-Runge, K., & Newbury-Birch, D. (2022). A systematic review and meta-analysis on the prevalence of mental disorders among children and adolescents in Europe. *European Child & Adolescent Psychiatry*. 10.1007/s00787-022-02131-2 10.1007/s00787-022-02131-2 PMC9800241 36581685

[CR32] Skuse, D. H. (2007). Rethinking the nature of genetic vulnerability to autistic spectrum disorders. *TRENDS in Genetics,**23*(8), 387–395. 17630015 10.1016/j.tig.2007.06.003

[CR33] Simonoff, E., Pickles, A., Wood, N., Gringras, P., & Chadwick, O. (2007). ADHD Symptoms in Children With Mild Intellectual Disability. *Journal of the American Academy of Child & Adolescent Psychiatry,**46*(5), 591–600. 10.1097/chi.0b013e3180323330 17450050 10.1097/chi.0b013e3180323330

[CR34] Song, P., Zha, M., Yang, Q., Zhang, Y., Li, X., & Rudan, I. (2021). The prevalence of adult attention-deficit hyperactivity disorder: A global systematic review and meta-analysis. *Journal of Global Health,**11*, 04009. 10.7189/jogh.11.04009 33692893 10.7189/jogh.11.04009 PMC7916320

[CR35] Syriopoulou- Delli, C. K., & Papaefstathiou, E. (2020). Review of cluster analysis of phenotypic data in Autism Spectrum Disorders: Distinct subtypes or a severity gradient model? *International Journal of Developmental Disabilities,**66*(1), 13–21. 10.1080/20473869.2018.1542561 10.1080/20473869.2018.1542561 PMC8115451 34141364

[CR36] Trillingsgaard, A., & Østergaard, J. R. (2004). Autism in Angelman Syndrome: An Exploration of Comorbidity. *Autism,**8*(2), 163–174. 10.1177/1362361304042720 15165432 10.1177/1362361304042720

[CR37] Writzl, K., & Knegt, A. C. (2013). 6p21.3 microdeletion involving the SYNGAP1 gene in a patient with intellectual disability, seizures, and severe speech impairment. *American Journal of Medical Genetics Part A.,**161*(7), 1682–1685. 10.1002/ajmg.a.35930 10.1002/ajmg.a.35930 23687080

[CR38] Zollino, M., Gurrieri, F., Orteschi, D., Marangi, G., Leuzzi, V., & Neri, G. (2011). Integrated analysis of clinical signs and literature data for the diagnosis and therapy of a previously undescribed 6p21.3 deletion syndrome. *European Journal of Human Genetics,**19*(2), 2. 10.1038/ejhg.2010.172 10.1038/ejhg.2010.172 PMC3025798 21119708

