# Supplementary material for: Profiling Autism and Attention Deficit Hyperactivity Disorder Traits in Children with SYNGAP1-Related Intellectual Disability
Source: J Autism Dev Disord. 2023 Dec 6;55(1):297–309. doi: 10.1007/s10803-023-06162-9 (PMC11802683; doi:10.1007/s10803-023-06162-9)
Supplement: Supplementary file 1 — Supplementary file1 (DOCX 22 KB) [file 10803_2023_6162_MOESM1_ESM.docx]

**Supplementary analysis**

*Sample characteristics*

Of those who had received a SYNGAP1-ID diagnosis, 6 had received an additional diagnosis of ASD alone, and 11 an additional diagnosis of epilepsy alone. One individual had received dual additional diagnoses of ADHD and epilepsy. Five individuals had received dual additional diagnoses for both ASD and epilepsy. One individual had been diagnosed with ASD, ADHD and epilepsy. Six SYNGAP1-ID individuals had not received a diagnosis of either ASD, ADHD or epilepsy.

*Medication use*

In total, 8 of those with SYNGAP1-ID reported to be on no medication whilst we were unable to obtain medication information from a further two individuals. Of those in the SYNGAP1-ID group taking medication, one individual was on medication (Atomoxetine) for ADHD. Many of those on medication reported being on more than one. See supplementary table 1 for a full breakdown of the potentially psychotropic medications reportedly used by those with SYNGAP1-ID.

**Supplementary table 1.** Medication use of SYNGAP1-ID group.

| **Medication** | **N used** |
| --- | --- |
| Sodium valproate | 5 |
| Levetiracetam | 5 |
| Clobazam | 5 |
| Ethosuximide | 4 |
| Lamotrigine | 4 |
| Melatonin | 4 |
| Lansoprazole | 2 |
| Midazolam | 1 |
| Risperodone | 1 |
| Lamotrigine | 1 |
| Atomoxetine | 1 |
| Flupentixol | 1 |
| Fluoxetine | 1 |
| CBD oil | 1 |
| Hyoscine | 1 |
| Potassium citrate | 1 |
| Promethazine | 1 |

*Correlations between measures*

We examined associations between the SRS, SCQ and the Conners for those with SYNGAP1-ID. Bivariate correlation analysis highlighted a number of positive associations between the measures. In particular, those that scored high on the Conners hyperactivity scale were also likely to score high for SRS total (r(19) = 0.603, p=0.006), and SCQ total score (r(19) 0.602, p=0.006). Further, the Conners Global Index showed a positive association with SRS total (r(19) = 0.564, p=0.01), and SCQ total score (r(19) 0.596, p=0.007), SRS RRB (r(19) 0.545, p=0.01) and SRS RRB (r(19) 0.580, p=0.009). Alongside this, the Conners DSM-5 hyperactive scale showed a significant positive association with SRS total score (r(19) = 0.528, p=0.02),and the SCQ total score (r(19) 0.639, p=0.003). However, after correcting for multiple comparisons only the positive association between SRS RRB and Conners DSM-5 hyperactivity (r(19)= 0.680, p=0.001) survived, whilst the positive association between SRS RRB and Conners hyperactivity showed a trend towards significance (r(19)=0.665, p=0.002).
